# The Influence of Carrier Oils on the Antimicrobial Activity and Cytotoxicity of Essential Oils

**DOI:** 10.1155/2019/6981305

**Published:** 2019-01-14

**Authors:** Ané Orchard, Guy Kamatou, Alvaro M. Viljoen, Namita Patel, Patricia Mawela, Sandy F. van Vuuren

**Affiliations:** ^1^University of the Witwatersrand, Faculty of Health Sciences, Department of Pharmacy and Pharmacology, 7 York Road, Parktown 2193, South Africa; ^2^Tshwane University of Technology, Faculty of Science, Department of Pharmaceutical Sciences, Private Bag X680, Pretoria 0001, South Africa; ^3^SAMRC Herbal Drugs Research Unit, Department of Pharmaceutical Sciences, Private Bag X680, Pretoria 0001, South Africa

## Abstract

The topical use of essential oils requires dilution into a carrier oil; however, scientific evidence regarding the antimicrobial efficacy and cytotoxicity when a carrier oil is combined with an essential oil is lacking. This study sets out to determine the antimicrobial activity and cytotoxicity of 23 essential oils combined with six known carrier oils. Gas chromatography-mass spectrometry/flame ionization detector (GC-MS/FID) was used to characterize the methyl esters of the carrier oils. The antimicrobial activity of the carrier oils alone and in combination with the essential oils was investigated using the broth microdilution assay against 11 skin pathogens and the cytotoxicity was determined using the brine shrimp lethality assay. The interactive profiles of the combinations for both antimicrobial activity and the cytotoxicity were analysed and calculated using the fractional inhibitory concentration index (ΣFIC). The carrier oils demonstrated no antimicrobial antagonism when combined with the essential oils and the overall cytotoxicity of the majority of the combinations was decreased. The carrier oils that could be identified as enhancing the antimicrobial activity and decreasing the cytotoxicity were* Aloe vera* Mill. and* Simmondsia chinensis* C.K.Schneid (Jojoba oil), with an overall reduction in essential oil cytotoxicity of 87.5% at 24 hrs and 85% at 48 hrs by* A. vera*. Five of the essential oils (when diluted in* A. vera* and* S. chinensis* carrier oils) demonstrated enhanced antimicrobial activity against pathogens such as* Brevibacterium epidermidis*,* B. linens,* and* P. aeruginosa *with MIC values ranging from 0.09 to 0.50 mg/mL (and ΣFIC 0.14-0.39). The study could conclude that the carrier oils are complementary to essential oil formulations, mostly reducing cytotoxicity and in some cases enhancing the antimicrobial activity.

## 1. Introduction

Essential oils are popular in complementary medicine and often used to treat dermatological conditions. A common example is* Melaleuca alternifolia* Cheel. (tea tree), which is known worldwide as an anti-infective essential oil and is used in many commercial acne products. These products are considered as a “safe” alternative and a preferred holistic treatment by consumers, but what has not been considered is the common use with a carrier oil. Recent findings of the antimicrobial activity of essential oils against skin pathogens prompted further research into the combined use with carrier oils [1, 2]. Essential oils are rarely used undiluted when applied directly to the skin [3]. Neat oils have been shown to cause skin irritation such as contact dermatitis [4]. They are thus blended into a base before application to the skin. This combination of essential oil and carrier oil not only dilutes the essential oils but also is believed to make the essential oils less toxic on the skin, slow down the evaporation rate, and increase essential oil absorption [3]. Increased absorption is achieved as carrier oils are composed of molecules which make them closely related to sebum (the skin's own natural oil). Possible bases that essential oils can be diluted into include creams, gels, or carrier oils (also known as vegetable or fixed oils) [5]. It has, however, been found that a base can influence the overall antimicrobial activity of the oil [6]. Yet, there is a paucity of information relating to the effects of carrier oils on the antimicrobial activity and cytotoxicity of essential oils [7]; thus this study investigates this void with special emphasis on oils implicated in dermatology and interactions affecting skin pathogens.

## 2. Materials and Methods

### 2.1. Oil Selection

The selection of six carrier oils was based on the aroma-therapeutic literature available to the layman as reviewed by Orchard and van Vuuren [7], where the most frequently cited carrier oils for dermatological use and those claimed as having antimicrobial activity were selected. The selection of the 23 essential oils was made based on a range of activity, representative of noteworthy, moderate, and poor antimicrobial activity [1], so that an overall understanding could be drawn of carrier oil effects against different antimicrobial potencies. Consideration was also given to essential oils with poor activity, as, in combination, they may demonstrate synergy. Popular essential oils that are well known to the layman, such as* M. alternifolia* and* Lavandula angustifolia* Mill. (lavender), were also included (See supplementary data for selection (available here)). The carrier oils were obtained from Essentia. Essential oils were procured from Givaudan (Dubendorf, Switzerland), Robertet (Grasse, France), Burgess and Finch, Prana Monde, Essentia, and Scatters Oils (all Gauteng, South Africa).

### 2.2. Chemical Analysis of the Carrier Oils

The chemical profiles of the essential oils have been previously investigated and reported [1]. The chemical characterization of the carrier oils required separation via the induction of volatility by methylating and conversion of the carrier oils into fatty acid methyl esters (FAMEs). This required the addition of 1.0 mL of hexane into 0.1 mL of the carrier oil. Thereafter, 1.0 mL sodium methoxide (1.6 g of NaOH in 50.0 mL of methanol) solution was added, and this mixture was vortexed for 30 s. This solution was then centrifuged at 1200 rpm and incubated at room temperature for 10 min to allow for the separation of the clear solution of FAMEs. The methyl esters were then transferred to a vial for analysis [8]. The FAME composition of each carrier oil was analysed by gas chromatography-mass spectrometry/flame ionization detector (GC-MS/FID). The GC-MS/FID (Agilent 6860 N GC system) (Agilent Technologies, USA) was coupled directly to a 5973 mass selective detector (MSD) equipped with a 5% phenyl, polymethyl siloxane column (30 m× 250 mm i.d. × 0.25 *μ*m film thickness). A volume of 1.0 *μ*L was injected (using a split ratio of 1:50) with an autosampler at 24.79 psi and an inlet temperature of 250°C. The GC oven temperature was initially 60°C for 10 min, rising to 250°C at a rate of 4°C/min. Helium was used as a carrier gas at a constant flow of 1.0 mL/min. Spectra were obtained on the electron impact at 70 eV, scanning from 35 to 450* m/z*. The percentage composition of the individual components was quantified by integration measurements using flame ionization detection (FID, 250°C) while the identification was based on the total ion chromatogram (TIC) using ChemStation software. Component identification was made by using available FAMEs reference compounds in combination with library searching of the Mass Finder®, Flavor®, and NIST libraries [9].

### 2.3. Preparation of Cultures

The 11 microorganisms tested were either from the American Type Culture Collection (ATCC) or Deutsche Summlung von Mikrooganismen (DSM) strains. The skin pathogens included reference strains of the wound pathogens* Staphylococcus aureus* ATCC 25923 (including two antibiotic resistant strains, methicillin resistant* S. aureus* (MRSA) ATCC 43300, and gentamicin-methicillin resistant* S. aureus* (GMRSA) ATCC 33592) and two Gram-negative bacteria (*Pseudomonas aeruginosa *ATCC 27858,* Escherichia coli *ATCC 25922), odour inducing bacteria (*Brevibacterium agri* ATCC 51663,* B. epidermidis* DSM 20660,* B. linens* DSM 20425), acne pathogens (*S. epidermidis* ATCC 2223, and* Propionibacterium acnes *ATCC 11827), and the yeast* Candida albicans* ATCC 10231.* Candida albicans* was inoculated into Tryptone Soya broth (TSB) (Oxoid) and incubated at 37°C for 48 hrs. The fastidious pathogen,* P. acnes* was grown in Thioglycolate broth (TGB) (Oxoid) and incubated under anaerobic conditions using a CO_2_ incubator (8.4% CO_2_) for seven days at 37°C.* Brevibacterium linens* required inoculation into TSB and incubation at 30°C for four days, and the remaining bacterial cultures were also all inoculated into TSB for 24 hrs at 37°C. All cultures were streaked onto Tryptone Soya agar (Oxoid) and incubated to confirm purity. The University of the Witwatersrand Human Research Ethics Committee (Reference W-CJ-131026-3) granted a waiver for the use of these microorganisms.

### 2.4. Minimum Inhibitory Concentration (MIC)

The broth microdilution method was determined as being the preferred method for investigating carrier oil activity [7]. The carrier oils alone and in combination with essential oils were tested in 1:1 ratios using the broth microdilution assay as detailed in [2]. After the aseptic preparation of 100 *μ*L of sterile, distilled water was added to each of the 96 wells of a microtitre plate, followed by 100 *μ*L of the carrier oil, or 50 *μ*L essential oil with 50 *μ*L carrier oil for the combinations, to the first row. This was serially diluted descending down the microtitre plate columns. Antimicrobial susceptibility was confirmed with the inclusion of 0.01 mg/mL ciprofloxacin (Sigma Aldrich®) (for bacteria) or 0.10 mg/mL amphotericin B (Sigma Aldrich®) (for* C. albicans*). A negative control of 32.0 mg/mL water in acetone was included to ensure the antimicrobial activity was not as a result of the solvent. The respective growth media of each pathogen were also included to ensure the support of microbial growth. After the preparation of an approximate inoculum concentration of 1 × 10^6^ colony forming units per mL (CFU mL^−1^) for each microorganism, 100 *μ*L was added to each well. Sterile adhesive sealing film was used to seal each microtitre plate and the plates were incubated accordingly. After incubation, 40 *μ*L of 0.04% (w/v)* p*-iodonitrotetrazolium violet solution (INT) (Sigma Aldrich®) was added to each well. A colour change of the indicator to pink or purple was indicative of microbial growth; thus the lowest concentration displaying no growth was taken as the minimum inhibitory concentration (MIC). Each sample and combination was tested in triplicate and the mean taken. The MIC values were recorded and, for the combinations, the fractional inhibitory concentration index (ΣFIC) was calculated [10].

The ΣFIC values of the combinations were calculated using the following equations:

(1) $$\begin{array}{c} \begin{array}{c} FIC\left(i\right)=\frac{Value\;\;of\;\left(a\;*\right)\;combined\;\;with\;\;value\;\;of\;\;\left(b\;*\right)}{Value\;\;of\;\left(a\right)\;independently} \end{array}\\ FIC\left(\;ii\;\right)=\frac{Value\;\;of\;\left(b\right)\;combined\;\;with\;\;value\;\;of\;\;\left(a\right)}{Value\;\;of\;\left(b\right)\;independently} \end{array}$$

*∗*where (a) is either the MIC value of the first essential oil in the combination and (b) is the MIC value of the carrier oil.

The FIC index could then be calculated: ΣFIC = FIC (i) + FIC (ii). If ΣFIC for the combination was ≤ 0.5 synergy was indicated, a ΣFIC of > 0.5 and ≤ 1.0 is indicative of an additive interaction, > 1.0 - ≤ 4.0 indicated indifference, and > 4.0 indicated antagonism [10].

### 2.5. Brine Shrimp Lethality Assay

The preparation of artificial sea water was undertaken by dissolving 16 g of Tropic Marine® sea salt in 500 mL of sterile distilled water. Dried* Artemia franciscana* (brine shrimp) eggs (Ocean Nutrition™) (0.5 g) were added to the salt water and aerated with a rotary pump (Kiho). A constant source of warmth and light was provided with a lamp (220–240 V). The incubation of the eggs under these conditions was at 25°C for 18-24 hrs.

The cytotoxicity evaluation was conducted in 48-well microtitre plates, where 400 *μ*L salt water containing 40-60 live brine shrimp was added to each well. The test samples at a concentration of 1.00 mg/mL (carrier oils, essential oils, or the combination of both) was then added (400 *μ*L) to each well in triplicate. To ensure that the artificial sea water supported the growth and survival of the brine shrimp by mimicking their natural environment, 32.0 g/L artificial sea water was included as a negative control, along with 2% dimethyl sulfoxide (DMSO), which was used as the solvent for the samples. Potassium dichromate (Sigma Aldrich), which is a known toxic compound, was used as a positive control at a concentration of 1.60 mg/mL. The plates were viewed after 24 and 48 hrs under a light microscope (Olympus) at 40× magnification and the dead brine shrimp counted. Each sample and combination was tested in triplicate and the mean taken. A lethal dose of acetic acid (Saarchem; 100% (v/v); 50.0 *μ*L) was thereafter added to each well and a final count of dead brine shrimp undertaken [11]. The percentage mortality was calculated using the following equation:

(2) $$\begin{array}{c} \begin{array}{c} \%\;\;Mortality=\frac{dead\;\;shrimp\;\;at\;\;24\;\;hours\;\left(or\;\;48\;\;hours\right)\;\left(\text{before}\;acetic\;\;acid\right)-dead\;\;shrimp\;\left(time\;\;0\right)}{dead\;\;shrimp\;\left(\text{after}\;acetic\;\;acid\right)}\times 100 \end{array} \end{array}$$

Biological cytotoxicity was considered for a percentage mortality of 50% or greater [12]. The fractional inhibitory concentration index (ΣFIC) was calculated for the combinations to determine the overall effect off the carrier oils on the essential oil cytotoxicity. The ΣFIC ranges were classified as follows: synergy for values ≤ 0.5, where there is a substantial decrease in cytotoxicity, additive interactions for a ΣFIC range between > 0.5 and ≤ 1.0, indifference for a ΣFIC range of between > 1.0 and ≤ 4.0, and > 4.0 indicated antagonism where an increase in cytotoxicity was observed for the combination.

## 3. Results and Discussion

### 3.1. *Chemical Analysis*

The chemical composition of the essential oils has been previously reported [1] and is included in Table 1. It is known that the chemical composition of essential oils may vary due to differences in chemotype, storage, harvesting, or plant origin [13–15]. For example, the sample of* Thymus vulgaris* from this study has a lower content of thymol (18.9%) than reported in previous studies (44.7-48.9%) [16–18] and the* Laurus nobilis* sample used in this study contains myrcene and chavicol, as opposed to high levels of 1,8-cineole (35.5-42.3%) as previously reported [19, 20]. Five out of the six carrier oils contained both linoleic acid and oleic acid methyl esters (Table 2).* Aloe vera *Mill. (aloe vera)*, Calendula officinalis *L. (calendula), and* Hypericum perforatum* L. (St John's wort) are characterized by high levels of linoleic acid methyl ester (59.8-69.9%).* Persea americana* Mill. (avocado) and* Prunus armeniaca* Blanco (apricot kernel) are dominated by a high percentage of oleic acid, methyl ester (60.7-69.5%).* Simmondsia chinensis *C.K.Schneid (jojoba) was found to be different from the other carrier oils with eicosenoic acid (55.6%) being the major fatty acid.* Aloe vera* and* P. americana *oils also contained vitamin E.

### 3.2. Antimicrobial Activity

The antimicrobial activity of the essential oils has previously been reported [1]. The carrier oils, overall, displayed poor antimicrobial activity (Table 2). The only bacterial species that was somewhat inhibited by the carrier oils was* P. aeruginosa* and* B. epidermidis *at 1.00 mg/mL for selected carrier oils. The poor antimicrobial activity is not surprising as they are not expected to exhibit antimicrobial activity [7] but rather provide other properties against skin conditions such as anti-inflammatory, anti-oxidant, or an increase in collagen synthesis [7]. From an antimicrobial perspective, another two studies also reported poor antimicrobial activity of* S. chinensis* [21]. When studied in combination, however, De Prijck, Peeters, and Nelis [21] reported an increase in antimicrobial activity of the* S. chinensis* carrier oil and* M. alternifolia* essential oil combination tested against* E. coli*,* S. aureus,* and* P. aeruginosa*. Unfortunately, the diffusion assay was utilised, making comparison with the current study difficult and somewhat inaccurate due to the inherent flaws associated with oils and agar diffusion assays. This study, however, could report on moderate antimicrobial activity (2.00 mg/mL) for this combination. This is the first study to report on* S. chinensis* against MRSA, GMRSA, acne, and bromodosis pathogens.

*Aloe vera* gel has been reported to display antimicrobial activity [14]. The antimicrobial activity of the carrier oil, however, is reported for the first time in this study. As with this study,* P. armeniaca* has also previously been reported to display poor antimicrobial activity (>2.00% v/v) against* E. coli*,* P. aeruginosa*,* S. aureus,* and* H. perforatum *was previously shown to display poor to no antimicrobial activity against* S. aureus* [15]. However, to the best of our knowledge, the antimicrobial activity of* C. officinalis* and* P. americana* carrier oils are reported here for the first time against all 11 pathogens. Although not expected to display antimicrobial activity, the observed antimicrobial activity of the carrier oils may likely be attributed to the free fatty acids, such as linoleic acid and oleic acids, which individually have displayed antimicrobial activity against the Gram-positive micro-organisms such as* P. acnes* and* S. aureus* at high concentrations [16].

**Figure 1:**
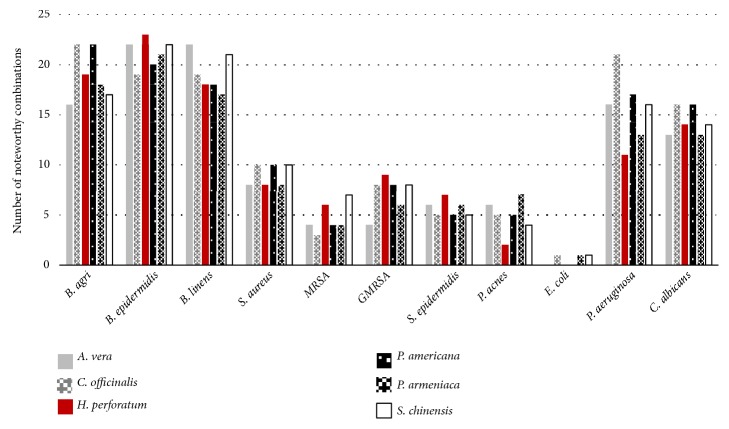
Summary of the antimicrobial activity of essential oil: carrier oil combinations activity.

**Figure 2:**
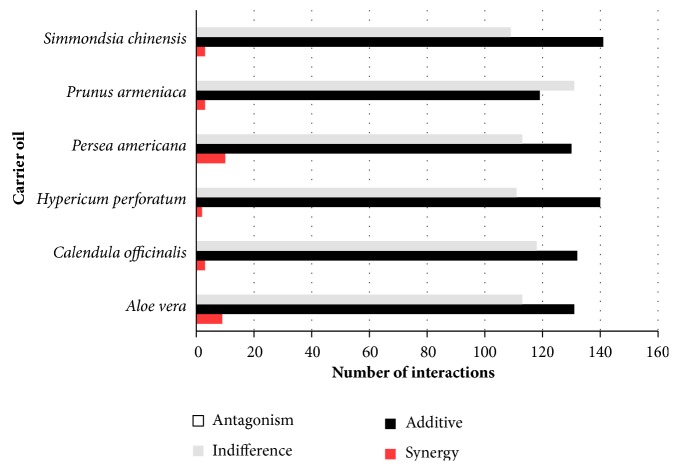
Summary of interactive profiles of essential oil with carrier oil combinations.

The antimicrobial activity of the essential oils combined with the carrier oils (1518 combinations consisting of 23 essential oils combined with six different carrier oils, against 11 pathogens involved in skin infections), along with the interactive profiles of the combinations, is displayed in Table 3.  Figure 1 summarises the antimicrobial activity of the essential oils combined with each carrier oil, and Figure 2 summarises the interactive profiles of all the studied combinations. The majority of the combinations were found to be additive (52.2%), and 45.8% were found to be indifferent. Synergy was also identified in 30 combinations, and, encouragingly, the antimicrobial activity of the essential oils, when diluted with the carrier oils, did not result in antagonism. The carrier oils that most frequently exhibited promising synergy were* P. americana* (ten combinations), followed by* A. vera* (nine combinations).

While dilution of the essential oils with carrier oils in most cases resulted in a slight decrease in antimicrobial activity, it was interesting to note that the antimicrobial activity of several combinations was actually enhanced when tested against selected pathogens. This was observed for combinations with* A. vera* and* S. chinensis*, which increased antimicrobial activity against* B. epidermidis*,* B. linens,* and* P. aeruginosa*. It raises the question as to whether the permeating properties exhibited by* S. chinensis* carrier oil and* A. vera* gel (carrier oil research is lacking) are able to enhance permeation into the bacterial cell wall [17, 18], an area of investigation which should be encouraged. The carrier oil* H. perforatum* increased the antimicrobial activity of several essential oils against* B. epidermidis. Calendula officinalis *and* P. americana* increased the essential oil activity against* P. aeruginosa*. What is remarkable, though, is that no antimicrobial antagonism was observed between any of the 1518 carrier oils: essential oil combinations investigated, validating the aroma-therapeutic use of combining carrier oils with essential oils.

### 3.3. Cytotoxicity Studies

The percentage cytotoxicity of each carrier oil is shown in Table 2. None of the carrier oils were found to be toxic. In fact,* A. vera*,* C. officinalis *and* P. americana* were found to show no trace of cytotoxicity after 24 hrs when tested in the brine shrimp lethality assay. The low cytotoxicity shown by* S. chinensis* and* C. officinalis* has also previously been observed [17, 19]. The cytotoxicity of* H. perforatum, P. americana,* and* P. armeniaca* carrier oils, however, is reported for the first time in this study.

The cytotoxicity profiles of the controls and the essential oils alone and in combination with the carrier oils are displayed in Table 4. The majority of the essential oils were found to be cytotoxic at 24 and 48 hrs, even at a low concentration of 1.00 mg/mL. The essential oils that were found to be nontoxic after 48 hrs were* Helichrysum italicum* (immortelle),* Laurus nobilis* (bay), and* Vetiveria zizanioides* (vetiver). A previous report on the cytotoxicity of* L. nobilis* is limited to its potency against* Camptomyia corticalis* [20].* Helichrysum italicum* and* V. zizanioides* were also investigated previously [20], with no reported cytotoxicity.

*Aloe vera* and* S. chinensis* reduced the cytotoxicity of 14 essential oils after 24 hrs, followed by* H. perforatum*,* P. americana,* and* P. armeniaca* that decreased the cytotoxicity of 13 essential oils, and lastly* C. officinalis* decreased the cytotoxicity of 11 oils. Considering that essential oils may be applied daily to the skin, this highlights* A. vera* and* S. chinensis* as the carrier oils that would be most beneficial for topical use. Due to three of the carrier oils displaying no cytotoxicity at 24 hrs, the ΣFIC could not be calculated (FIC calculation requires an endpoint value); however, values could be calculated for 48 hr exposure. After 48 hrs, 18 out of 23 combinations of essential oils with* Aloe vera* resulted in a synergistic decrease in cytotoxicity. Seventeen synergistic interactions were observed with* H. perforatum* and 14 with* C. officinalis*. There is a statistical significant difference between the two periods (all* p* < 0.05).* Aloe vera* was the carrier oil that showed the most pronounced quenching of essential oil cytotoxicity at both 24 and 48 hrs. It draws attention to whether the vitamin E in the carrier oil may contribute towards this effect. Previous studies have shown vitamin E to decrease the cytotoxicity of medicines [22], and vitamin E derivative *α*-tocopherol has also been shown to decrease toxicity [23].

**Figure 3:**
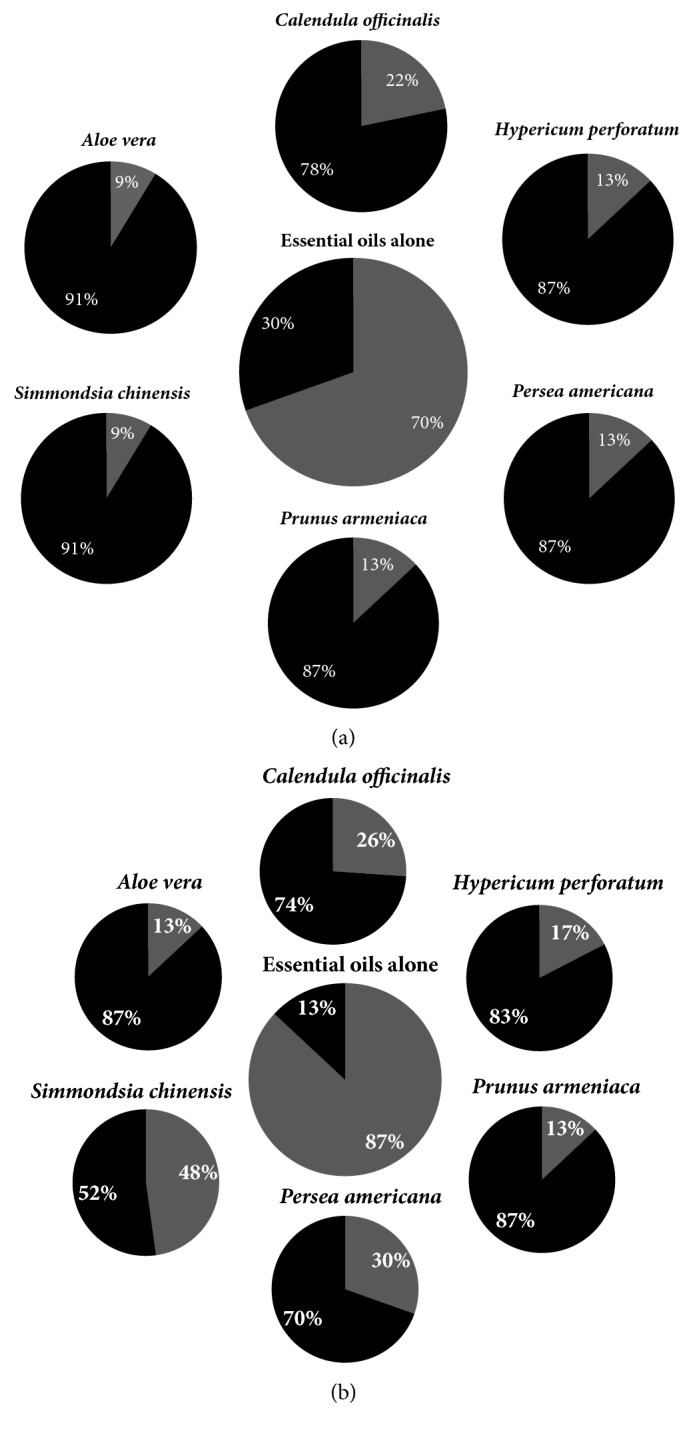
Summary of essential oil cytotoxicity alone and when in combination with each carrier oil (a, decrease in cytotoxicity after 24 hrs; b, decrease of cytotoxicity after 48 hrs). Non-toxic is shown as black and cytotoxic is shown as grey.

Figure 3 summarises the percentage of essential oils that were found to be toxic alone and the percentage of toxic essential oils once combined with carrier oils. At 24 hrs (a), 70% of the essential oils were toxic and this decreased to 9-22% once combined with the respective carrier oil. The essential oil cytotoxicity at 48 hrs (b) decreased from 87% down to 13-48% once combined with the respective carrier oil. The carrier oils that decrease the cytotoxicity from the essential oils the most was* A. vera* and* S. chinensis* at 24 hrs and* A. vera* and* P. armeniaca* at 48 hrs. Overall it can be noted that the carrier oils decreased the cytotoxicity of the essential oils.

Several essential oils were found to display high levels of cytotoxicity, with only half or less of the carrier oils decreasing their cytotoxicity. These include* Citrus bergamia* (bergamot),* Cymbopogon citratus* (lemongrass),* Syzygium aromaticum* (clove), and* Thymus vulgaris* (thyme).* Syzygium aromaticum* was also previously reported to display cytotoxicity against human fibroblasts [24] and only two carrier oils (*A. vera* and* S. chinensis*) decreased the cytotoxicity of this oil.* Citrus bergamia *is known to cause phototoxicity [25] but cytotoxicity could be decreased by* P. americana*,* P. armeniaca,* and* S. chinensis*; and* C. citratus* and* T. vulgaris *were also previously reported to display cytotoxicity [26, 27].* Thymus vulgaris* cytotoxicity was reduced by* P. americana* only, and* C. citratus *essential oil cytotoxicity was minimised by* A. vera, H. perforatum,* and* P. armeniaca*.

*Cymbopogon martinii* (palmarosa) is an essential oil, often recommended for acne, and* S. chinensis* is the carrier oil used for acne treatment, as previously reviewed [13]; however, the combination of* C. martinii* with* S. chinensis* was found to remain toxic at 24 hrs.

In practice, smaller quantities of essential oils are blended into larger quantities of carrier oils and it is suggested that the carrier oil blend should consist of only 1-3% of essential oil:carrier oil mix [28]. As the combinations investigated in this study were performed at a 1:1 ratio, it may be possible that the essential oil cytotoxicity would decrease further once diluted into a higher concentration of carrier oils.

## 4. Conclusion

This study can conclude that carrier oils exert a positive effect over essential oil cytotoxicity without causing antagonism of the antimicrobial activity. Synergy was frequently observed and a reduction of essential oil cytotoxicity after 48 hrs was mostly found.* Aloe vera* and* S. chinensis* were identified as the carrier oils that caused the highest reduction of cytotoxicity and increased antimicrobial activity the most.* Aloe vera*,* S. chinensis, H. perforatum C. officinalis, *and* P. americana *were found to increase antimicrobial activity of several essential oils against respective pathogens, including* B. epidermidis*,* B. linens,* and* P. aeruginosa*. Additional studies could include different chemotypes as well as the essential oil:carrier oil combinations where carrier oils are used at a higher ratio, especially against the more toxic essential oils. The carrier oils are also presented as an option for use in dermatology to decrease cytotoxicity of medicines that cause skin irritation.

## Figures and Tables

**Table 1:** Essential oil main compounds.

Essential oil	GC-MS FID^*∗*^
*Cananga odorata *Hook.f. & Thomson (ylang ylang)	Benzyl acetate (30.5%)
	Linalyl acetate (27.7%)
	Methyl benzoate (10.3%)
	Methylanisole (10.9%)
*Cinnamomum zeylanicum *Blume (cinnamon leaf)	Eugenol (78.4%)
*Citrus bergamia* Risso (bergamot)	Limonene (39.1%)
	Linalyl acetate (9.3%)
	Linalyl formate (33.0%)
*Citrus reticulata* Blanco (mandarin)	Limonene (93.8%)
*Commiphora myrrha *Engl. (myrrh)	Furanoeusdema-1,3-diene (52.9%)
	Lindestrene (15.8%)
*Cymbopogon citratus *Stapf (lemongrass)	Limonene (11.7%)
	Neral (28.9%)
	Geranial (42.7%)
*Cymbopogon martinii *Stapf (palmarosa)	Geraniol (80.7%)
*Eucalyptus globulus *Labill. (eucalyptus)	1,8-Cineole (63.6%)
	*α*-Terpineol (10.5%)
*Helichrysum italicum* (Roth) G.Don (immortelle)	*β*-Pinene (7.6%)
	1,8-Cineole (19.4%)
	*β*-Caryophyllene (12.4%)
	*α*-Humulene (15.0%)
*Kunzea ericoides* (A.Rich.) Joy Thomps. (kanuka)	*α*-Pinene (52.9%)
	*p*-Cymene (11.9%)
*Laurus nobilis* L. (bay)	Eugenol (54.4%)
	Myrcene (18.5%)
	Chavicol (11.5%)
*Lavandula angustifolia* Mill. (lavender)	Linalyl acetate (35.6%)
	Linalool (32.8%)
	*β*-Caryophyllene (10.2%)
*Leptospermum scoparium* J.R.Forst. and G.Forst (manuka)	Neral (23.0%)
	Geranial (35.0%)
	Citronellol (15.4%)
*Litsea cubeba *Pers. (may chang)	Geranial (44.6%)
	Neral (28.8%)
*Melaleuca alternifolia* Cheel (tea tree)	*γ*-Terpinene (16.6%)
	*p*-Cymene (9.6%)
	Terpinen-4-ol (44.6%)
*Melaleuca viridiflora *Gaertn. (niaouli)	Linalyl acetate (49.8%)
	Linalool (30.1%)
*Melissa officinalis *L. (lemon balm)	Citronellal (35.6%)
	Neral (7.1%)
	Terpinyl acetate (14.2%)
	*α*-Terpineol (7.3%)
	Geranial (8.4%)
	*cis*-Geraniol (7.2%)
*Pogostemon patchouli *Benth. (patchouli)	*β*-Patchoulene (38.3%)
	*α*-Bulnesene (13.0%)
	*α*-Guaiene (11.9%)
*Santalum album *L. (sandalwood)	*α*-Santalol (32.1%)
	*cis*-*α*-Santalol (11.3%)
	4,5,9,10-Dehydroisolongifolene (12.3%)
*Styrax benzoin* Dryand. (benzoin)	Benzyl acetate (7.2%)
	Ethyl cinnamate (5.7%)
	Dimethyl phthalate (72.2%)
*Syzygium aromaticum *(L.) Merr. & L.M.Perry (clove)	Eugenol (81.9%)
	Isoeugenol (13.1%)
*Thymus vulgaris *L. (thyme)	Thymol (18.9%)
	*γ*-Terpinene (7.2%)
	*p*-Cymene (41.0%)
*Vetiveria zizanioides *Stapf (vetiver)	*β*-Vetirenene (8.8%)
	Zizanol (12.8%)

^*∗*^Previously reported in Orchard, Sandasi, Kamatou, Viljoen, and van Vuuren [1]

**Table 2:** Antimicrobial activity, cytotoxicity and fatty acid composition of carrier oils.

**Carrier oil**	**Fatty acid composition**	**Antimicrobial activity (mg/mL)**											**Cytotoxicity ** ** (**%** mortality)**	
		***P. acnes*** **ATCC 11827**	***S. epi*** **ATCC 2223**	***S. aureus*** **ATCC 25924**	**MRSA ATCC 43300**	**GMRSA ATCC 33592**	***E. coli*** **ATCC 8739**	***P. aerug*** **ATCC 27858**	***B. agri*** **ATCC 51663**	***B. epi*** **DSM 20660**	***B. lin*** **DSM 20425**	***C. alb*** **ATCC 10231**	**24 hrs**	**48 hrs**
*Aloe vera *(aloe vera)	linoleic acid methyl ester (59.8%)	16.00	16.00	10.00	8.00	4.00	8.00	1.00	2.00	1.00	8.00	8.00	0.00	27.08
	oleic acid, methyl ester (23.0%)													
	palmitic acid methyl ester (8.9%)													
	stearic acid methyl ester (4.2%)													
	linolenic acid methyl ester (3.7%)													
	Vitamin E - 14 IU 46%													
*Calendula officinalis *(calendula)	linoleic acid methyl ester (66.8%)	3.00	16.00	4.00	5.60	7.00	4.00	1.50	2.00	2.00	8.00	16.00	0.00	28.35
	oleic acid, methyl ester (24.6%)													
	palmitic acid methyl ester (14.9%)													
	linolenic acid methyl ester (1.0%)													
*Hypericum perforatum *(St John's wort)	linoleic acid methyl ester (69.9%)	2.50	16.00	4.00	4.00	4.00	4.00	1.50	2.00	1.00	2.00	4.00	0.63	39.33
	oleic acid, methyl ester (19.0%)													
	stearic acid methyl ester (5.0%)													
	palmitic acid methyl ester (5.8%)													
	linolenic acid methyl ester (0.1%)													
*Persea americana *(avacado)	oleic acid, methyl ester (69.5%)	8.00	16.00	4.00	5.20	4.00	4.00	1.75	2.00	4.00	8.00	16.00	0.00	25.75
	palmitic acid methyl ester (14.9%)													
	linoleic acid methyl ester (9.2%)													
	palmitoleic acid methyl ester (5.5%)													
	linolenic acid methyl ester (0.6%)													
*Prunus armeniaca *(apricot kernel)	oleic acid, methyl ester (60.7%)	8.00	6.00	2.00	4.00	2.00	4.00	1.00	2.00	1.00	6.00	8.00	4.35	10.38
	linoleic acid methyl ester (27.0%)													
	palmitic acid methyl ester (5.7%)													
	palmitoleic acid methyl ester (4.7%)													
	stearic acid methyl ester (0.9%)													
	Vitamin E 13.2 mg/100g													
*Simmondsia chinensis *(jojoba)	11-eicosenoic acid, methyl ester (55.6%)	1.50	16.00	4.00	4.00	4.00	4.00	1.00	2.00	1.00	2.00	6.00	3.04	24.12
	palmitic acid methyl ester (15.9%)													

Antimicrobial controls	ciprofloxacin *µ*g/mL	0.78 x 10^−3^	0.25 x 10^−3^	0.5 x 10^−3^	0.78 x 10^−3^	0.31 x 10^−3^	0.08 x 10^−3^	0.31 x 10^−3^	0.16 x 10^−3^	0.26 x 10^−3^	0.63 x 10^−3^	n.a		
	amphotericin *µ*g/mL	^*∗*^n.a	n.a	n.a	n.a	n.a	n.a	n.a	n.a	n.a	n.a	6.25 x 10^−3^		
DMSO	mg/mL												5.26	19.34
Sea water	mg/mL												10.75	32.72
Potassium dichromate	mg/mL												99.44	100.00

^*∗*^n.a., not applicable. *S. epi*: *S. epidermidis*; *P. aerug*: *P. aeruginosa*; *B. epi*: *B. epidermidis*; *B. lin*: *B. linens*; *C. alb*: *C. albicans*.

**Table 3:** Antimicrobial activity (mg/mL) of essential oils combined with carrier oils against skin pathogens.

***B. agri *ATCC 51663**													
		*Aloe* *vera*		*Calendula* *officinalis*		*Hypericum* *perforatum*		*Persea* *americana*		*Prunus* *armeniaca*		*Simmondsia* *chinensis*	
Essential oil	**EO MIC**	**MIC**	Σ**FIC**	**MIC**	Σ**FIC**	**MIC**	Σ**FIC**	**MIC**	Σ**FIC**	**MIC**	Σ**FIC**	**MIC**	Σ**FIC**

*Cananga odorata *(ylang ylang)	**0.50**	1.00^**∗**^	1.25	**1.00**	1.25	2.00	2.50	**1.00**	1.25	**1.00**	1.25	**1.00**	1.25
*Cinnamomum zeylanicum *(cinnamon)	**0.50**	**1.00**	1.25	**1.00**	1.25	**0.50**	0.63	**0.50**	0.63	**1.00**	1.25	**1.00**	1.25
*Citrus bergamia* (bergamot)	**1.00**	2.00	1.50	**1.00**	0.75	2.00	1.50	**1.00**	0.75	2.00	1.50	2.00	1.50
*Citrus reticulata* (mandarin)	**1.00**	**1.00**	0.75	**1.00**	0.75	**1.00**	0.75	**0.50**	***0.38†***	**1.00**	0.75	1.50	1.13
*Commiphora myrrha *(myrrh)	**0.38**	**0.38**	0.60	**0.44**	0.69	**0.38**	0.60	**0.38**	0.60	**0.50**	0.78	**0.35**	0.55
*Cymbopogon citratus *(lemongrass)	**0.25**	**1.00**	2.25	**0.50**	1.13	**0.50**	1.13	**0.50**	1.13	**0.50**	1.13	**0.50**	1.13
*Cymbopogon martinii *(palmarosa)	**0.38**	**1.00**	1.57	**0.50**	0.78	**0.50**	0.78	**0.33**	0.52	**0.75**	1.17	**1.00**	1.57
*Eucalyptus globulus* (eucalyptus)	**0.50**	2.00	2.50	1.50	1.88	**1.00**	1.25	2.00	2.50	**1.00**	1.25	2.00	2.50
*Helichrysum italicum *(immortelle)	**0.50**	**1.00**	1.25	**1.00**	1.25	**1.00**	1.25	**0.75**	0.94	**1.00**	1.25	**1.00**	1.25
*Kunzea ericoides* (kanuka)	**1.00**	2.00	1.50	**1.00**	0.75	2.00	1.50	**1.00**	0.75	2.00	1.50	2.00	1.50
*Laurus nobilis* (bay)	**0.50**	**1.00**	1.25	**0.50**	0.63	**1.00**	1.25	**0.50**	0.63	**1.00**	1.25	**0.50**	0.63
*Lavandula angustifolia* (lavender)	**1.00**	1.50	1.13	**1.00**	0.75	**1.00**	0.75	**1.00**	0.75	1.50	1.13	2.00	1.50
*Leptospermum scoparium* (manuka)	**0.38**	**0.50**	0.78	**0.50**	0.78	**0.50**	0.78	**0.50**	0.78	**0.50**	0.78	**0.50**	0.78
*Litsea cubeba *(may chang)	**0.13**	**0.80**	3.28	**0.50**	2.05	**0.50**	2.05	**0.50**	2.05	**0.50**	2.05	**0.75**	3.07
*Melaleuca alternifolia* (tea tree)	**1.00**	1.50	1.13	**1.00**	0.75	**1.00**	0.75	**1.00**	0.75	**1.00**	0.75	1.50	1.13
*Melaleuca viridiflora* (niaouli)	**1.00**	3.00	2.25	**1.00**	0.75	2.00	1.50	**1.00**	0.75	2.00	1.50	**1.00**	0.75
*Melissa officinalis* (lemon balm)	**1.00**	**1.00**	0.75	**0.75**	0.56	**1.00**	0.75	**0.50**	***0.38***	**1.00**	0.75	**1.00**	0.75
*Pogostemon patchouli* (patchouli)	**0.09**	**0.60**	3.48	**0.19**	1.10	**0.13**	0.73	**0.25**	1.45	**0.60**	3.48	**0.50**	2.90
*Santalum album *(sandalwood)	**0.31**	0.38	0.70	**0.31**	0.58	**0.30**	0.56	**0.31**	0.58	**0.31**	0.58	**0.31**	0.58
*Styrax benzoin *(benzoin)	**0.50**	1.00	1.25	**1.00**	1.25	**1.00**	1.25	**1.00**	1.25	1.50	1.88	**1.00**	1.25
*Syzygium aromaticum* (clove)	**0.50**	1.00	1.25	**1.00**	1.25	**0.75**	0.94	**0.50**	0.63	**0.75**	0.94	**1.00**	1.25
*Thymus vulgaris *(thyme)	**0.50**	2.00	2.50	**1.00**	1.25	**1.00**	1.25	**1.00**	1.25	**1.00**	1.25	**1.00**	1.25
*Vetiveria zizanioides *(vetiver)	**0.05**	0.25	2.56	**0.25**	2.56	**0.25**	2.56	**0.25**	2.56	**0.38**	3.84	**0.25**	2.56

***B. epidermidis *DSM 20660**													
		*Aloe* *vera*		*Calendula* *officinalis*		*Hypericum* *perforatum*		*Persea* *americana*		*Prunus* *armeniaca*		*Simmondsia* *chinensis*	
Essential oil	**EO MIC**	**MIC**	Σ**FIC**	**MIC**	Σ**FIC**	**MIC**	Σ**FIC**	**MIC**	Σ**FIC**	**MIC**	Σ**FIC**	**MIC**	Σ**FIC**

*Cananga odorata *(ylang ylang)	**0.50**	**0.50**	0.75	**1.00**	1.25	**0.50**	0.75	**1.00**	1.13	**0.50**	0.75	**0.13**	***0.19***
*Cinnamomum zeylanicum *(cinnamon)	1.50	**0.75**	0.63	**1.00**	0.58	**0.75**	0.63	1.20	0.55	**0.75**	0.63	**0.75**	0.63
*Citrus bergamia* (bergamot)	**0.50**	**0.50**	0.75	**1.00**	1.25	**1.00**	1.50	**1.00**	1.13	**0.50**	0.75	**0.75**	1.13
*Citrus reticulata* (mandarin)	1.50	**1.00**	0.83	2.00	1.17	**0.75**	0.63	2.00	0.92	**0.75**	0.63	**0.75**	0.63
*Commiphora myrrha *(myrrh)	**0.25**	**0.25**	0.63	**0.75**	1.69	**0.25**	0.63	**0.50**	1.06	**0.25**	0.63	**0.25**	0.63
*Cymbopogon citratus *(lemongrass)	**0.50**	**0.50**	0.75	**1.00**	1.25	**0.50**	0.75	**1.00**	1.13	**0.50**	0.75	**0.50**	0.75
*Cymbopogon martinii *(palmarosa)	**0.50**	**1.00**	1.50	**1.00**	1.25	**0.50**	0.75	**1.00**	1.13	**0.50**	0.75	**0.50**	0.75
*Eucalyptus globulus* (eucalyptus)	**0.50**	2.00	3.00	2.00	2.50	**1.00**	1.50	**0.25**	***0.28***	2.50	3.75	2.00	3.00
*Helichrysum italicum *(immortelle)	**0.50**	**0.50**	0.75	2.00	2.50	**0.50**	0.75	**1.00**	1.13	**0.50**	0.75	**0.50**	0.75
*Kunzea ericoides* (kanuka)	**1.00**	**0.75**	0.75	**1.00**	0.75	**0.75**	0.75	**1.00**	0.63	**0.75**	0.75	**0.75**	0.75
*Laurus nobilis* (bay)	**0.25**	**0.38**	0.94	**0.50**	1.13	**0.50**	1.25	**0.50**	1.06	**0.50**	1.25	**0.50**	1.25
*Lavandula angustifolia* (lavender)	**1.00**	**0.75**	0.75	**1.00**	0.75	**0.75**	0.75	**1.00**	0.63	**0.75**	0.75	**0.75**	0.75
*Leptospermum scoparium* (manuka)	**1.00**	**0.75**	0.75	**1.00**	0.75	**0.75**	0.75	**1.00**	0.63	**0.75**	0.75	**0.75**	0.75
*Litsea cubeba *(may chang)	**1.00**	**0.75**	0.75	**1.00**	0.75	**0.75**	0.75	2.00	1.25	**0.75**	0.75	**1.00**	1.00
*Melaleuca alternifolia* (tea tree)	**0.50**	**1.00**	1.50	**1.00**	1.25	**1.00**	1.50	**1.00**	1.13	2.00	3.00	**1.00**	1.50
*Melaleuca viridiflora* (niaouli)	**1.00**	**0.75**	0.75	**1.00**	0.75	**0.75**	0.75	**1.00**	0.63	**0.75**	0.75	**0.75**	0.75
*Melissa officinalis* (lemon balm)	**1.00**	**0.75**	0.75	1.50	1.13	**0.75**	0.75	**1.00**	0.63	**0.75**	0.75	**0.75**	0.75
*Pogostemon patchouli* (patchouli)	**0.50**	**0.09**	***0.14***	**1.00**	1.25	**0.50**	0.75	**1.00**	1.13	**0.50**	0.75	**0.40**	0.60
*Santalum album *(sandalwood)	**0.25**	**0.25**	0.63	**0.25**	0.56	**0.25**	0.63	**0.25**	0.53	**0.25**	0.63	**0.25**	0.63
*Styrax benzoin *(benzoin)	**0.50**	**0.50**	0.75	**1.00**	1.25	**0.50**	0.75	**1.00**	1.13	**0.50**	0.75	**0.50**	0.75
*Syzygium aromaticum* (clove)	**0.38**	**0.50**	0.91	**0.50**	0.78	**0.50**	0.91	**0.50**	0.72	**0.50**	0.91	**0.50**	0.91
*Thymus vulgaris *(thyme)	**0.50**	**0.50**	0.75	**1.00**	1.25	**0.50**	0.75	**1.00**	1.13	**1.00**	1.50	**0.50**	0.75
*Vetiveria zizanioides *(vetiver)	**0.19**	**0.19**	0.60	**0.50**	1.44	**0.19**	0.60	**0.50**	1.38	**0.25**	0.78	**0.19**	0.60

***B. linens *DSM 20425**													
		*Aloe* *vera*		*Calendula* *officinalis*		*Hypericum* *perforatum*		*Persea* *americana*		*Prunus* *armeniaca*		*Simmondsia* *chinensis*	
Essential oil	**EO MIC**	**MIC**	Σ**FIC**	**MIC**	Σ**FIC**	**MIC**	Σ**FIC**	**MIC**	Σ**FIC**	**MIC**	Σ**FIC**	**MIC**	Σ**FIC**

*Cananga odorata *(ylang ylang)	**1.00**	**1.00**	0.56	**1.00**	0.56	**0.75**	0.56	1.50	0.84	**1.00**	0.58	**1.00**	0.75
*Cinnamomum zeylanicum *(cinnamon)	**0.50**	**1.00**	1.06	**1.00**	1.06	**0.45**	0.56	**0.50**	0.53	**0.50**	0.54	**0.75**	0.94
*Citrus bergamia* (bergamot)	2.00	**0.50**	***0.16***	1.70	0.53	1.10	0.55	1.75	0.55	1.75	0.58	1.20	0.60
*Citrus reticulata* (mandarin)	**1.00**	**1.00**	0.56	**1.00**	0.56	**1.00**	0.75	2.00	1.13	**1.00**	0.58	**0.75**	0.56
*Commiphora myrrha *(myrrh)	**0.38**	**0.50**	0.69	**0.38**	0.52	**0.38**	0.60	**0.38**	0.52	**0.38**	0.52	**0.38**	0.60
*Cymbopogon citratus *(lemongrass)	**0.50**	**0.50**	0.53	**1.00**	1.06	**0.50**	0.63	**0.50**	0.53	**0.50**	0.54	**0.45**	0.56
*Cymbopogon martinii *(palmarosa)	**0.75**	**0.75**	0.55	**1.00**	0.73	**0.60**	0.55	**1.00**	0.73	**0.75**	0.56	**0.75**	0.69
*Eucalyptus globulus* (eucalyptus)	**1.00**	**1.00**	0.56	2.00	1.13	**1.00**	0.75	**1.00**	0.56	**1.00**	0.58	**1.00**	0.75
*Helichrysum italicum *(immortelle)	**0.50**	**0.50**	0.53	**1.00**	1.06	**0.50**	0.63	**1.00**	1.06	**0.50**	0.54	**0.63**	0.78
*Kunzea ericoides* (kanuka)	2.00	**0.50**	***0.16***	1.75	0.55	1.50	0.75	1.75	0.55	2.00	0.67	1.25	0.63
*Laurus nobilis* (bay)	**0.50**	**0.50**	0.53	**0.75**	0.80	**1.00**	1.25	**0.50**	0.53	**0.75**	0.81	**0.50**	0.63
*Lavandula angustifolia* (lavender)	**1.00**	**1.00**	0.56	**1.00**	0.56	1.17	0.88	**1.00**	0.56	2.00	1.17	**1.00**	0.75
*Leptospermum scoparium* (manuka)	**0.50**	**0.75**	0.80	**1.00**	1.06	**0.50**	0.63	**1.00**	1.06	**0.63**	0.68	**0.63**	0.78
*Litsea cubeba *(may chang)	**0.50**	**1.00**	1.06	**1.00**	1.06	**0.50**	0.63	**1.00**	1.06	**0.50**	0.54	**1.00**	1.25
*Melaleuca alternifolia* (tea tree)	1.50	1.50	0.59	1.50	0.59	**1.00**	0.58	1.30	0.51	1.50	0.63	**1.00**	0.58
*Melaleuca viridiflora* (niaouli)	**1.00**	**1.00**	0.56	**1.00**	0.56	**0.75**	0.56	**1.00**	0.56	**1.00**	0.58	**0.75**	0.56
*Melissa officinalis* (lemon balm)	**1.00**	**1.00**	0.56	**1.00**	0.56	1.50	1.13	**1.00**	0.56	1.25	0.73	**0.75**	0.56
*Pogostemon patchouli* (patchouli)	**0.75**	**0.75**	0.55	**0.75**	0.55	**0.75**	0.69	**0.75**	0.55	**0.75**	0.56	**0.75**	0.69
*Santalum album *(sandalwood)	**0.25**	**0.50**	1.03	**0.25**	0.52	**0.50**	1.13	**0.25**	0.52	**0.50**	1.04	**0.50**	1.13
*Styrax benzoin *(benzoin)	**0.75**	**0.25**	***0.18***	**1.00**	0.73	**0.75**	0.69	**0.75**	0.55	**0.75**	0.56	**0.63**	0.57
*Syzygium aromaticum* (clove)	**0.50**	**0.50**	0.53	**0.75**	0.80	**0.50**	0.63	**1.00**	1.06	**0.50**	0.54	**0.50**	0.63
*Thymus vulgaris *(thyme)	**1.00**	**1.00**	0.56	**1.00**	0.56	1.50	1.13	**1.00**	0.56	1.50	0.88	**0.75**	0.56
*Vetiveria zizanioides *(vetiver)	**0.19**	**1.00**	2.69	**0.50**	1.35	**0.33**	0.96	**0.50**	1.35	**0.50**	1.36	**0.19**	0.54

***S. aureus *ATCC 25924**													
		*Aloe* *vera*		*Calendula* *officinalis*		*Hypericum* *perforatum*		*Persea* *americana*		*Prunus* *armeniaca*		*Simmondsia* *chinensis*	
Essential oil	**EO MIC**	**MIC**	Σ**FIC**	**MIC**	Σ**FIC**	**MIC**	Σ**FIC**	**MIC**	Σ**FIC**	**MIC**	Σ**FIC**	**MIC**	Σ**FIC**

*Cananga odorata *(ylang ylang)	2.00	2.00	0.60	2.00	0.75	2.00	0.75	2.00	0.75	2.00	1.00	2.00	0.75
*Cinnamomum zeylanicum *(cinnamon)	**0.75**	**1.00**	0.72	**1.00**	0.79	**1.00**	0.79	**1.00**	0.79	**1.00**	0.92	**1.00**	0.79
*Citrus bergamia* (bergamot)	2.00	2.00	0.60	2.00	0.75	2.00	0.75	2.00	0.75	2.00	1.00	3.00	1.13
*Citrus reticulata* (mandarin)	2.00	2.67	0.80	3.00	1.13	2.00	0.75	4.00	1.50	2.00	1.00	2.00	0.75
*Commiphora myrrha *(myrrh)	**1.00**	**1.00**	0.55	**1.00**	0.63	**1.00**	0.63	**1.00**	0.63	**1.00**	0.75	**1.00**	0.63
*Cymbopogon citratus *(lemongrass)	**1.00**	**1.00**	0.55	**1.00**	0.63	2.00	1.25	**1.00**	0.63	**1.00**	0.75	**1.00**	0.63
*Cymbopogon martinii *(palmarosa)	**0.50**	1.50	1.58	**1.00**	1.13	1.50	1.69	**1.00**	1.13	**1.00**	1.25	2.00	2.25
*Eucalyptus globulus* (eucalyptus)	2.00	4.00	1.20	8.00	3.00	2.00	0.75	8.00	3.00	4.00	2.00	5.00	1.88
*Helichrysum italicum *(immortelle)	**1.00**	2.00	1.10	3.00	1.88	2.00	1.25	3.00	1.88	2.00	1.50	2.00	1.25
*Kunzea ericoides* (kanuka)	2.00	2.00	0.60	2.00	0.75	2.00	0.75	2.00	0.75	5.00	2.50	2.00	0.75
*Laurus nobilis* (bay)	**1.00**	**1.00**	0.55	**1.00**	0.63	**1.00**	0.63	**1.00**	0.63	2.00	1.50	**1.00**	0.63
*Lavandula angustifolia* (lavender)	4.00	3.00	0.53	2.50	0.63	2.50	0.63	4.00	1.00	2.00	0.75	2.50	0.63
*Leptospermum scoparium* (manuka)	**1.00**	**1.00**	0.55	**1.00**	0.63	**1.00**	0.63	**1.00**	0.63	**1.00**	0.75	**1.00**	0.63
*Litsea cubeba *(may chang)	**1.00**	**1.00**	0.55	**1.00**	0.63	1.50	0.94	**1.00**	0.63	**1.00**	0.75	**1.00**	0.63
*Melaleuca alternifolia* (tea tree)	2.00	2.00	0.60	2.00	0.75	3.00	1.13	2.00	0.75	2.00	1.00	2.00	0.75
*Melaleuca viridiflora* (niaouli)	**1.00**	2.00	1.10	2.00	1.25	2.00	1.25	2.00	1.25	2.00	1.50	2.00	1.25
*Melissa officinalis* (lemon balm)	**1.00**	2.00	1.10	2.00	1.25	3.00	1.88	2.00	1.25	2.00	1.50	2.00	1.25
*Pogostemon patchouli* (patchouli)	**0.50**	2.00	2.10	**1.00**	1.13	2.00	2.25	1.50	1.69	2.00	2.50	**1.00**	1.13
*Santalum album *(sandalwood)	**0.50**	**0.50**	0.53	**0.50**	0.56	**0.50**	0.56	**0.50**	0.56	**1.00**	1.25	**0.50**	0.56
*Styrax benzoin *(benzoin)	**1.00**	2.00	1.10	2.00	1.25	**1.00**	0.63	2.00	1.25	2.00	1.50	2.00	1.25
*Syzygium aromaticum* (clove)	**0.88**	1.50	0.93	1.50	1.04	**1.00**	0.69	**1.00**	0.69	1.50	1.23	**1.00**	0.69
*Thymus vulgaris *(thyme)	**1.00**	2.00	1.10	2.00	1.25	3.00	1.88	2.00	1.25	4.00	3.00	2.00	1.25
*Vetiveria zizanioides *(vetiver)	**0.50**	**0.75**	0.79	**0.50**	0.56	**1.00**	1.13	**0.50**	0.56	**1.00**	1.25	**0.50**	0.56

**MRSA ATCC 43300**													
		*Aloe* *vera*		*Calendula* *officinalis*		*Hypericum* *perforatum*		*Persea* *americana*		*Prunus* *armeniaca*		*Simmondsia* *chinensis*	
Essential oil	**EO MIC**	**MIC**	Σ**FIC**	**MIC**	Σ**FIC**	**MIC**	Σ**FIC**	**MIC**	Σ**FIC**	**MIC**	Σ**FIC**	**MIC**	Σ**FIC**

*Cananga odorata *(ylang ylang)	4.00	3.00	0.56	4.00	0.86	3.00	0.75	4.00	0.88	2.50	0.63	3.00	0.75
*Cinnamomum zeylanicum *(cinnamon)	1.50	2.00	0.79	2.00	0.85	2.00	0.92	2.00	0.86	2.00	0.92	1.20	0.55
*Citrus bergamia* (bergamot)	4.00	4.00	0.75	4.00	0.86	2.50	0.63	8.00	1.77	4.00	1.00	4.00	1.00
*Citrus reticulata* (mandarin)	4.00	6.00	1.13	4.00	0.86	6.00	1.50	4.00	0.88	8.00	2.00	4.00	1.00
*Commiphora myrrha *(myrrh)	**0.50**	**1.00**	1.06	**1.00**	1.09	**1.00**	1.13	**1.00**	1.10	**1.00**	1.13	**1.00**	1.13
*Cymbopogon citratus *(lemongrass)	**1.00**	2.00	1.13	2.00	1.18	**1.00**	0.63	**1.00**	0.60	2.00	1.25	**1.00**	0.63
*Cymbopogon martinii *(palmarosa)	1.50	2.00	0.79	2.00	0.85	1.50	0.69	3.00	1.29	2.00	0.92	1.50	0.69
*Eucalyptus globulus* (eucalyptus)	2.00	6.00	1.88	8.00	2.71	4.00	1.50	8.00	2.77	4.00	1.50	4.00	1.50
*Helichrysum italicum *(immortelle)	2.00	4.00	1.25	6.00	2.04	3.00	1.13	3.00	1.04	5.00	1.88	4.00	1.50
*Kunzea ericoides* (kanuka)	5.00	4.00	0.65	4.00	0.76	4.00	0.90	3.00	0.59	4.00	0.90	3.00	0.68
*Laurus nobilis* (bay)	**1.00**	2.00	1.13	2.00	1.18	2.00	1.25	2.00	1.19	2.00	1.25	**1.00**	0.63
*Lavandula angustifolia* (lavender)	2.00	4.00	1.25	4.00	1.36	4.00	1.50	4.00	1.38	4.00	1.50	4.00	1.50
*Leptospermum scoparium* (manuka)	**1.00**	2.00	1.13	3.00	1.77	**1.00**	0.63	2.00	1.19	1.50	0.94	**1.00**	0.63
*Litsea cubeba *(may chang)	**0.50**	**1.00**	1.06	**1.00**	1.09	**1.00**	1.13	2.00	2.19	**1.00**	1.13	**1.00**	1.13
*Melaleuca alternifolia* (tea tree)	2.00	3.00	0.94	4.00	1.36	2.00	0.75	4.00	1.38	4.00	1.50	2.00	0.75
*Melaleuca viridiflora* (niaouli)	2.00	4.00	1.25	4.00	1.36	4.00	1.50	8.00	2.77	4.00	1.50	2.00	0.75
*Melissa officinalis* (lemon balm)	**1.00**	2.00	1.13	4.00	2.36	2.00	1.25	4.00	2.38	2.00	1.25	2.00	1.25
*Pogostemon patchouli* (patchouli)	**1.00**	3.00	1.69	4.00	2.36	2.00	1.25	4.00	2.38	2.00	1.25	2.00	1.25
*Santalum album *(sandalwood)	**0.50**	**0.50**	0.53	**1.00**	1.09	**0.50**	0.56	**1.00**	1.10	**0.75**	0.84	**0.50**	0.56
*Styrax benzoin *(benzoin)	2.00	2.00	0.63	2.00	0.68	2.00	0.75	4.00	1.38	2.00	0.75	2.00	0.75
*Syzygium aromaticum* (clove)	**1.00**	2.00	1.13	2.00	1.18	2.00	1.25	2.00	1.19	2.00	1.25	1.50	0.94
*Thymus vulgaris *(thyme)	2.00	2.00	0.63	4.00	1.36	2.00	0.75	4.00	1.38	2.00	0.75	2.00	0.75
*Vetiveria zizanioides *(vetiver)	**0.50**	**1.00**	1.06	2.00	2.18	**0.50**	0.56	**1.00**	1.10	**1.00**	1.13	**0.75**	0.84

**GMRSA ATCC 33592**													
		*Aloe* *vera*		*Calendula* *officinalis*		*Hypericum* *perforatum*		*Persea* *americana*		*Prunus* *armeniaca*		*Simmondsia* *chinensis*	

Essential oil	**EO MIC**	**MIC**	Σ**FIC**	**MIC**	Σ**FIC**	**MIC**	Σ**FIC**	**MIC**	Σ**FIC**	**MIC**	Σ**FIC**	**MIC**	Σ**FIC**

*Cananga odorata *(ylang ylang)	4.00	4.00	1.00	4.00	0.79	3.00	0.75	4.00	1.00	2.00	0.75	2.50	0.63
*Cinnamomum zeylanicum *(cinnamon)	**0.13**	**1.00**	3.97	**1.00**	3.92	**1.00**	3.97	**1.00**	3.97	**0.75**	3.07	**1.00**	3.97
*Citrus bergamia* (bergamot)	6.00	4.00	0.83	2.00	***0.31***	4.00	0.83	3.00	0.63	4.00	1.33	4.00	0.83
*Citrus reticulata* (mandarin)	2.40	2.00	0.67	6.00	1.68	4.00	1.33	4.00	1.33	6.00	2.75	3.00	1.00
*Commiphora myrrha *(myrrh)	**0.25**	**0.50**	1.06	**0.25**	0.52	**0.25**	0.53	**0.25**	0.53	**0.75**	1.69	**0.25**	0.53
*Cymbopogon citratus *(lemongrass)	**0.75**	2.00	1.58	**1.00**	0.74	**1.00**	0.79	**1.00**	0.79	1.50	1.38	2.00	1.58
*Cymbopogon martinii *(palmarosa)	**1.00**	2.00	1.25	2.00	1.14	3.00	1.88	2.00	1.25	2.00	1.50	2.00	1.25
*Eucalyptus globulus* (eucalyptus)	2.00	2.00	0.75	3.00	0.96	3.00	1.13	2.00	0.75	3.00	1.50	4.00	1.50
*Helichrysum italicum *(immortelle)	1.50	3.00	1.38	2.00	0.81	2.00	0.92	2.00	0.92	3.00	1.75	2.00	0.92
*Kunzea ericoides* (kanuka)	4.00	4.00	1.00	2.00	***0.39***	3.00	0.75	2.50	0.63	4.00	1.50	3.00	0.75
*Laurus nobilis* (bay)	**1.00**	1.50	0.94	1.50	0.86	**1.00**	0.63	**1.00**	0.63	1.50	1.13	**1.00**	0.63
*Lavandula angustifolia* (lavender)	2.00	4.00	1.50	2.00	0.64	2.00	0.75	2.00	0.75	4.00	2.00	4.00	1.50
*Leptospermum scoparium* (manuka)	**1.00**	2.00	1.25	2.00	1.14	**1.00**	0.63	2.00	1.25	**1.00**	0.75	**1.00**	0.63
*Litsea cubeba *(may chang)	**0.50**	1.50	1.69	**1.00**	1.07	**1.00**	1.13	**1.00**	1.13	**1.00**	1.25	**1.00**	1.13
*Melaleuca alternifolia* (tea tree)	2.00	2.00	0.75	4.00	1.29	2.00	0.75	4.00	1.50	3.00	1.50	2.00	0.75
*Melaleuca viridiflora* (niaouli)	2.00	4.00	1.50	4.00	1.29	4.00	1.50	4.00	1.50	3.00	1.50	3.00	1.13
*Melissa officinalis* (lemon balm)	**1.00**	3.00	1.88	2.00	1.14	2.00	1.25	2.00	1.25	2.00	1.50	2.00	1.25
*Pogostemon patchouli* (patchouli)	**0.25**	1.75	3.72	**1.00**	2.07	1.80	3.83	1.80	3.83	1.50	3.38	1.80	3.83
*Santalum album *(sandalwood)	**0.25**	**0.50**	1.06	**0.50**	1.04	**0.25**	0.53	**0.25**	0.53	**0.38**	0.84	**0.25**	0.53
*Styrax benzoin *(benzoin)	2.00	2.00	0.75	2.00	0.64	2.00	0.75	2.00	0.75	2.00	1.00	2.00	0.75
*Syzygium aromaticum* (clove)	**1.00**	1.50	0.94	**1.00**	0.57	**1.00**	0.63	**1.00**	0.63	1.50	1.13	**1.00**	0.63
*Thymus vulgaris *(thyme)	**1.00**	4.00	2.50	4.00	2.29	2.00	1.25	4.00	2.50	4.00	3.00	2.00	1.25
*Vetiveria zizanioides *(vetiver)	**0.13**	**0.50**	1.99	**0.75**	2.94	**0.50**	1.99	**0.25**	0.99	**0.50**	2.05	**0.38**	1.49

***S. epidermidis *ATCC 2223**													
		*Aloe* *vera*		*Calendula* *officinalis*		*Hypericum* *perforatum*		*Persea* *americana*		*Prunus* *armeniaca*		*Simmondsia* *chinensis*	
Essential oil	**EO MIC**	**MIC**	Σ**FIC**	**MIC**	Σ**FIC**	**MIC**	Σ**FIC**	**MIC**	Σ**FIC**	**MIC**	Σ**FIC**	**MIC**	Σ**FIC**

*Cananga odorata *(ylang ylang)	2.00	2.00	0.56	2.00	0.56	2.00	0.56	1.50	***0.42***	8.00	2.67	6.00	1.69
*Cinnamomum zeylanicum *(cinnamon)	**1.00**	2.00	1.06	1.50	0.80	2.00	1.06	2.00	1.06	2.00	1.17	2.00	1.06
*Citrus bergamia* (bergamot)	3.00	3.00	0.59	2.00	***0.40***	3.00	0.59	4.00	0.79	4.00	1.00	4.00	0.79
*Citrus reticulata* (mandarin)	4.00	8.00	1.25	4.00	0.63	4.00	0.63	3.50	0.55	8.00	1.67	12.00	1.88
*Commiphora myrrha *(myrrh)	**0.50**	**1.00**	1.03	3.00	3.09	**1.00**	1.03	2.00	2.06	**0.75**	0.81	**1.00**	1.03
*Cymbopogon citratus *(lemongrass)	**1.00**	**1.00**	0.53	**1.00**	0.53	**1.00**	0.53	2.00	1.06	**1.00**	0.58	**1.00**	0.53
*Cymbopogon martinii *(palmarosa)	**1.00**	**1.00**	0.53	2.00	1.06	1.50	0.80	1.50	0.80	2.00	1.17	2.00	1.06
*Eucalyptus globulus* (eucalyptus)	2.00	6.00	1.69	6.00	1.69	3.00	0.84	6.00	1.69	4.00	1.33	6.00	1.69
*Helichrysum italicum *(immortelle)	2.00	2.00	0.56	3.00	0.84	2.00	0.56	3.00	0.84	2.75	0.92	2.00	0.56
*Kunzea ericoides* (kanuka)	2.00	6.00	1.69	4.00	1.13	4.00	1.13	4.00	1.13	6.00	2.00	2.00	0.56
*Laurus nobilis* (bay)	**1.00**	2.00	1.06	2.00	1.06	**1.00**	0.53	2.00	1.06	2.00	1.17	2.00	1.06
*Lavandula angustifolia* (lavender)	2.00	2.00	0.56	2.00	0.56	4.00	1.13	2.00	0.56	2.00	0.67	2.00	0.56
*Leptospermum scoparium* (manuka)	1.00	**1.00**	0.53	**1.00**	0.53	**1.00**	0.53	**1.00**	0.53	**1.00**	0.58	**1.00**	0.53
*Litsea cubeba *(may chang)	**0.75**	1.50	1.05	**1.00**	0.70	**1.00**	0.70	**1.00**	0.70	**1.00**	0.75	1.50	1.05
*Melaleuca alternifolia* (tea tree)	4.00	3.50	0.55	3.50	0.55	8.00	1.25	3.50	0.55	3.00	0.63	3.50	0.55
*Melaleuca viridiflora* (niaouli)	2.00	2.00	0.56	4.00	1.13	2.00	0.56	2.00	0.56	4.00	1.33	4.00	1.13
*Melissa officinalis* (lemon balm)	**1.00**	2.00	1.06	2.00	1.06	3.00	1.59	2.00	1.06	2.00	1.17	2.00	1.06
*Pogostemon patchouli* (patchouli)	**0.25**	1.75	3.55	1.50	3.05	1.75	3.55	1.50	3.05	1.75	3.65	1.50	3.05
*Santalum album *(sandalwood)	**0.13**	**0.25**	0.97	**0.63**	2.42	**0.50**	1.94	**0.38**	1.45	**0.50**	1.96	**0.38**	1.45
*Styrax benzoin *(benzoin)	3.00	3.00	0.59	3.00	0.59	4.00	0.79	3.00	0.59	6.00	1.50	4.00	0.79
*Syzygium aromaticum* (clove)	**1.00**	2.00	1.06	2.00	1.06	2.00	1.06	2.00	1.06	2.00	1.17	1.50	0.80
*Thymus vulgaris *(thyme)	**0.75**	2.00	1.40	1.50	1.05	3.00	2.09	**1.00**	0.70	2.00	1.50	2.00	1.40
*Vetiveria zizanioides *(vetiver)	**0.13**	**1.00**	3.88	**0.75**	2.91	**0.50**	1.94	**0.75**	2.91	**0.50**	1.96	**0.50**	1.94

***P. acnes *ATCC 11827**													
		*Aloe* *vera*		*Calendula* *officinalis*		*Hypericum* *perforatum*		*Persea* *americana*		*Prunus* *armeniaca*		*Simmondsi* *chinensis*	
Essential oil	**EO MIC**	**MIC**	Σ**FIC**	**MIC**	Σ**FIC**	**MIC**	Σ**FIC**	**MIC**	Σ**FIC**	**MIC**	Σ**FIC**	**MIC**	Σ**FIC**

*Cananga odorata *(ylang ylang)	2.00	2.00	0.56	8.00	3.33	2.00	0.90	2.00	0.63	2.00	0.63	6.00	3.50
*Cinnamomum zeylanicum *(cinnamon)	**1.00**	2.00	1.06	**1.00**	0.67	2.00	1.40	1.50	0.84	**1.00**	0.56	1.50	1.25
*Citrus bergamia* (bergamot)	2.00	2.00	0.56	6.50	2.71	8.00	3.60	8.00	2.50	2.00	0.63	3.00	1.75
*Citrus reticulata* (mandarin)	**0.50**	3.50	3.61	3.00	3.50	2.00	2.40	3.00	3.19	2.00	2.13	2.00	2.67
*Commiphora myrrha *(myrrh)	**0.50**	**0.50**	0.52	2.00	2.33	**0.50**	0.60	2.00	2.13	**1.00**	1.06	**1.00**	1.33
*Cymbopogon citratus *(lemongrass)	**0.50**	**1.00**	1.03	**1.00**	1.17	2.00	2.40	**1.00**	1.06	**1.00**	1.06	**1.00**	1.33
*Cymbopogon martinii *(palmarosa)	**1.00**	1.50	0.80	1.50	1.00	**1.00**	0.70	2.00	1.13	**1.00**	0.56	**1.00**	0.83
*Eucalyptus globulus* (eucalyptus)	2.00	4.00	1.13	6.00	2.50	2.00	0.90	8.00	2.50	6.00	1.88	5.00	2.92
*Helichrysum italicum *(immortelle)	**1.00**	2.00	1.06	5.00	3.33	4.00	2.80	**0.75**	***0.42***	2.00	1.13	2.00	1.67
*Kunzea ericoides* (kanuka)	2.00	2.00	0.56	2.00	0.83	6.00	2.70	4.00	1.25	3.00	0.94	3.00	1.75
*Laurus nobilis* (bay)	**1.00**	2.00	1.06	2.00	1.33	3.00	2.10	2.00	1.13	1.50	0.84	1.50	1.25
*Lavandula angustifolia* (lavender)	2.00	2.00	0.56	2.00	0.83	2.00	0.90	4.00	1.25	1.50	***0.47***	2.00	1.17
*Leptospermum scoparium* (manuka)	**0.55**	**1.00**	0.94	**1.00**	1.08	2.00	2.22	2.00	1.94	2.00	1.94	2.00	2.48
*Litsea cubeba *(may chang)	**0.88**	1.50	0.90	**1.00**	0.73	2.00	1.54	**1.00**	0.63	**1.00**	0.63	1.50	1.35
*Melaleuca alternifolia* (tea tree)	2.00	4.00	1.13	2.00	0.83	3.00	1.35	2.00	0.63	6.00	1.88	6.00	3.50
*Melaleuca viridiflora* (niaouli)	**1.00**	2.00	1.06	2.00	1.33	2.00	1.40	5.00	2.81	2.00	1.13	2.00	1.67
*Melissa officinalis* (lemon balm)	**1.00**	2.00	1.06	2.00	1.33	2.00	1.40	2.00	1.13	2.00	1.13	2.00	1.67
*Pogostemon patchouli* (patchouli)	**0.50**	**1.00**	1.03	2.00	2.33	1.50	1.80	2.00	2.13	**1.00**	1.06	2.50	3.33
*Santalum album *(sandalwood)	**0.69**	2.00	1.51	2.00	1.78	2.00	1.85	**0.60**	***0.47***	1.50	1.18	2.00	2.12
*Styrax benzoin *(benzoin)	**1.00**	2.00	1.06	4.00	2.67	3.00	2.10	3.00	1.69	7.00	3.94	2.00	1.67
*Syzygium aromaticum* (clove)	**0.50**	**1.00**	1.03	1.50	1.75	2.00	2.40	2.00	2.13	**1.00**	1.06	2.00	2.67
*Thymus vulgaris *(thyme)	**1.00**	1.50	0.80	2.00	1.33	2.00	1.40	2.00	1.13	1.50	0.84	2.00	1.67
*Vetiveria zizanioides *(vetiver)	**0.50**	**1.00**	1.03	**1.00**	1.17	2.00	2.40	**1.00**	1.06	1.50	1.59	**1.00**	1.33

***E. coli *ATCC 8739**													
		*Aloe* *vera*		*Calendula* *officinalis*		*Hypericum* *perforatum*		*Persea* *americana*		*Prunus* *armeniaca*		*Simmondsia* *chinensis*	
Essential oil	**EO MIC**	**MIC**	Σ**FIC**	**MIC**	Σ**FIC**	**MIC**	Σ**FIC**	**MIC**	Σ**FIC**	**MIC**	Σ**FIC**	**MIC**	Σ**FIC**

*Cananga odorata *(ylang ylang)	2.00	2.63	0.82	4.00	1.50	3.00	1.13	3.00	1.13	2.00	0.75	2.63	0.98
*Cinnamomum zeylanicum *(cinnamon)	2.00	2.00	0.63	2.00	**0.75**	2.00	0.75	2.00	0.75	2.00	0.75	3.00	1.13
*Citrus bergamia* (bergamot)	3.50	4.00	0.82	4.00	1.07	1.85	***0.50***	4.00	1.07	4.00	1.07	4.00	1.07
*Citrus reticulata* (mandarin)	2.40	4.00	1.08	4.00	1.33	8.00	2.67	1.50	***0.50***	4.00	1.33	2.00	0.67
*Commiphora myrrha *(myrrh)	2.00	1.70	0.53	2.00	**0.75**	1.40	0.53	3.00	1.13	1.40	0.53	1.40	0.53
*Cymbopogon citratus *(lemongrass)	**1.00**	1.50	0.84	2.00	1.25	2.00	1.25	2.00	1.25	2.00	1.25	2.00	1.25
*Cymbopogon martinii *(palmarosa)	**1.00**	2.00	1.13	2.00	1.25	2.00	1.25	2.00	1.25	2.00	1.25	2.00	1.25
*Eucalyptus globulus* (eucalyptus)	2.00	4.00	1.25	4.00	1.50	4.00	1.50	4.00	1.50	4.00	1.50	4.00	1.50
*Helichrysum italicum *(immortelle)	2.00	2.00	0.63	3.00	1.13	2.00	0.75	3.00	1.13	2.00	0.75	2.00	0.75
*Kunzea ericoides* (kanuka)	2.00	4.00	1.25	2.00	**0.75**	3.00	1.13	2.00	0.75	3.00	1.13	2.00	0.75
*Laurus nobilis* (bay)	**1.00**	3.00	1.69	2.00	1.25	4.00	2.50	2.00	1.25	2.00	1.25	2.00	1.25
*Lavandula angustifolia* (lavender)	2.00	4.00	1.25	2.00	**0.75**	3.00	1.13	2.00	0.75	4.00	1.50	2.00	0.75
*Leptospermum scoparium* (manuka)	1.75	2.00	0.70	3.00	1.23	4.00	1.64	2.00	0.82	2.00	0.82	2.00	0.82
*Litsea cubeba *(may chang)	**1.00**	3.00	1.69	2.00	1.25	2.00	1.25	2.00	1.25	2.00	1.25	2.00	1.25
*Melaleuca alternifolia* (tea tree)	2.00	4.00	1.25	2.00	**0.75**	4.00	1.50	2.00	0.75	4.00	1.50	4.00	1.50
*Melaleuca viridiflora* (niaouli)	2.00	4.00	1.25	2.00	**0.75**	2.00	0.75	2.00	0.75	4.00	1.50	4.00	1.50
*Melissa officinalis* (lemon balm)	2.00	4.00	1.25	2.00	**0.75**	6.00	2.25	2.00	0.75	4.00	1.50	2.00	0.75
*Pogostemon patchouli* (patchouli)	**0.88**	1.50	0.95	2.00	1.39	2.00	1.39	2.00	1.39	2.00	1.39	2.00	1.39
*Santalum album *(sandalwood)	**0.50**	2.00	2.13	**1.00**	1.13	2.00	2.25	2.00	2.25	**1.00**	1.13	2.00	2.25
*Styrax benzoin *(benzoin)	2.00	2.00	0.63	2.00	**0.75**	5.00	1.88	2.00	0.75	2.00	0.75	4.00	1.50
*Syzygium aromaticum* (clove)	**1.00**	2.00	1.13	2.00	1.25	2.00	1.25	2.00	1.25	2.00	1.25	2.00	1.25
*Thymus vulgaris *(thyme)	2.00	4.00	1.25	2.00	**0.75**	2.00	0.75	2.00	0.75	4.00	1.50	2.00	0.75
*Vetiveria zizanioides *(vetiver)	**0.60**	2.00	1.79	2.00	1.92	3.00	2.88	2.00	1.92	3.00	2.88	**1.00**	0.96

***P. aeruginosa *ATCC 27858**													
		*Aloe* *vera*		*Calendula* *officinalis*		*Hypericum* *perforatum*		*Persea* *americana*		*Prunus* *armeniaca*		*Simmondsia* *chinensis*	
Essential oil	**EO MIC**	**MIC**	Σ**FIC**	**MIC**	Σ**FIC**	**MIC**	Σ**FIC**	**MIC**	Σ**FIC**	**MIC**	Σ**FIC**	**MIC**	Σ**FIC**

*Cananga odorata *(ylang ylang)	**1.00**	**1.00**	1.00	**1.00**	0.83	**1.00**	0.83	**0.50**	***0.39***	**1.00**	1.00	2.00	2.00
*Cinnamomum zeylanicum *(cinnamon)	**0.75**	2.00	2.33	**1.00**	1.00	**1.00**	1.00	**0.75**	0.71	**1.00**	1.17	1.50	1.75
*Citrus bergamia* (bergamot)	1.50	**1.00**	0.83	**1.00**	0.67	**1.00**	0.67	2.00	1.24	1.50	1.25	**1.00**	0.83
*Citrus reticulata* (mandarin)	1.50	2.00	1.67	2.00	1.33	**1.00**	0.67	2.00	1.24	1.50	1.25	**1.00**	0.83
*Commiphora myrrha *(myrrh)	**1.00**	**1.00**	1.00	**1.00**	0.83	3.00	2.50	2.00	1.57	**1.00**	1.00	**1.00**	1.00
*Cymbopogon citratus *(lemongrass)	**1.00**	1.50	1.50	**1.00**	0.83	1.50	1.25	**1.00**	0.79	**1.00**	1.00	**1.00**	1.00
*Cymbopogon martinii *(palmarosa)	**1.00**	**1.00**	1.00	**1.00**	0.83	**1.00**	0.83	**1.00**	0.79	**0.58**	0.58	1.50	1.50
*Eucalyptus globulus* (eucalyptus)	2.00	2.00	1.50	2.00	1.17	**1.00**	0.58	3.00	1.61	2.00	1.50	**1.00**	0.75
*Helichrysum italicum *(immortelle)	**1.00**	**1.00**	1.00	**1.00**	0.83	1.50	1.25	**1.00**	0.79	2.00	2.00	**1.00**	1.00
*Kunzea ericoides* (kanuka)	**1.00**	**1.00**	1.00	**1.00**	0.83	2.00	1.67	**1.00**	0.79	3.00	3.00	**1.00**	1.00
*Laurus nobilis* (bay)	**1.00**	**1.00**	1.00	**1.00**	0.83	2.00	1.67	**1.00**	0.79	**1.00**	1.00	**1.00**	1.00
*Lavandula angustifolia* (lavender)	**1.00**	**1.00**	1.00	**1.00**	0.83	3.00	2.50	**1.00**	0.79	1.50	1.50	**1.00**	1.00
*Leptospermum scoparium* (manuka)	2.00	**1.00**	0.75	**1.00**	0.58	**1.00**	0.58	**1.00**	0.54	**1.00**	0.75	**1.00**	0.75
*Litsea cubeba *(may chang)	1.50	3.00	2.50	**1.00**	0.67	**1.00**	0.67	**1.00**	0.62	**1.00**	0.83	1.50	1.25
*Melaleuca alternifolia* (tea tree)	2.00	**1.00**	0.75	**1.00**	0.58	**1.00**	0.58	**1.00**	0.54	**1.00**	0.75	1.50	1.13
*Melaleuca viridiflora* (niaouli)	1.50	**1.00**	0.83	**1.00**	0.67	1.50	1.00	**1.00**	0.62	1.50	1.25	**1.00**	0.83
*Melissa officinalis* (lemon balm)	2.00	**1.00**	0.75	**1.00**	0.58	1.50	0.88	**1.00**	0.54	2.50	1.88	**1.00**	0.75
*Pogostemon patchouli* (patchouli)	**1.00**	**1.00**	1.00	**1.00**	0.83	2.00	1.67	2.00	1.57	**1.00**	1.00	**0.75**	0.75
*Santalum album *(sandalwood)	1.50	**1.00**	0.83	**1.00**	0.67	1.50	1.00	**1.00**	0.62	**1.00**	0.83	**1.00**	0.83
*Styrax benzoin *(benzoin)	**1.00**	**1.00**	1.00	**1.00**	0.83	**1.00**	0.83	**1.00**	0.79	2.00	2.00	1.50	1.50
*Syzygium aromaticum* (clove)	**1.00**	**1.00**	1.00	**1.00**	0.83	**1.00**	0.83	**0.50**	***0.39***	2.00	2.00	**1.00**	1.00
*Thymus vulgaris *(thyme)	**1.00**	2.00	2.00	**1.00**	0.83	1.50	1.25	2.00	1.57	**1.00**	1.00	**1.00**	1.00
*Vetiveria zizanioides *(vetiver)	**1.00**	2.00	2.00	**1.00**	0.83	2.00	1.67	**1.00**	0.79	**1.00**	1.00	1.50	1.50

***C. albicans *ATCC 10231**													
		*Aloe* *vera*		*Calendula* *officinalis*		*Hypericum* *perforatum*		*Persea* *americana*		*Prunus* *armeniaca*		*Simmondsia* *chinensis*	
Essential oil	**EO MIC**	**MIC**	Σ**FIC**	**MIC**	Σ**FIC**	**MIC**	Σ**FIC**	**MIC**	Σ**FIC**	**MIC**	Σ**FIC**	**MIC**	Σ**FIC**

*Cananga odorata *(ylang ylang)	**1.00**	1.50	0.84	**1.00**	0.53	1.50	0.94	**1.00**	0.53	**1.00**	0.56	1.50	0.88
*Cinnamomum zeylanicum *(cinnamon)	**0.50**	**0.50**	0.53	**0.50**	0.52	**0.75**	0.84	**0.50**	0.52	**0.75**	0.80	**0.75**	0.81
*Citrus bergamia* (bergamot)	**1.00**	**1.00**	0.56	**1.00**	0.53	2.00	1.25	**1.00**	0.53	3.00	1.69	**1.00**	0.58
*Citrus reticulata* (mandarin)	2.00	1.75	0.55	3.00	0.84	**1.00**	0.51	6.00	1.69	2.00	0.63	1.55	0.52
*Commiphora myrrha *(myrrh)	**1.00**	**1.00**	0.56	**1.00**	0.53	2.00	1.25	**1.00**	0.53	1.50	0.84	1.50	0.88
*Cymbopogon citratus *(lemongrass)	**0.63**	**0.75**	0.64	**0.63**	0.52	**1.00**	0.92	**0.63**	0.52	**1.00**	0.86	**0.75**	0.66
*Cymbopogon martinii *(palmarosa)	**0.75**	1.50	1.09	**1.00**	0.70	**1.00**	0.79	**1.00**	0.70	**1.00**	0.73	**1.00**	0.75
*Eucalyptus globulus* (eucalyptus)	**1.00**	1.50	0.84	4.00	2.13	**1.00**	0.63	1.50	0.80	6.00	3.38	**1.00**	0.58
*Helichrysum italicum *(immortelle)	2.00	1.75	0.55	2.00	0.56	**1.00**	0.51	1.80	0.51	2.00	0.63	1.55	0.52
*Kunzea ericoides* (kanuka)	1.50	**0.75**	***0.30***	1.50	0.55	**1.00**	***0.46***	1.40	0.51	3.00	1.19	**1.00**	***0.42***
*Laurus nobilis* (bay)	**1.00**	1.25	0.70	**1.00**	0.53	1.50	0.94	1.50	0.80	**1.00**	0.56	**1.00**	0.58
*Lavandula angustifolia* (lavender)	**1.00**	**0.80**	***0.45***	**1.00**	0.53	1.50	0.94	**1.00**	0.53	1.50	0.84	1.50	0.88
*Leptospermum scoparium* (manuka)	**1.00**	**1.00**	0.56	**1.00**	0.53	**1.00**	0.63	**1.00**	0.53	**1.00**	0.56	**0.95**	0.55
*Litsea cubeba *(may chang)	**0.50**	**1.00**	1.06	**0.50**	0.52	**1.00**	1.13	**0.50**	0.52	**1.00**	1.06	**1.00**	1.08
*Melaleuca alternifolia* (tea tree)	1.50	**0.83**	***0.33***	1.50	0.55	1.50	0.69	1.50	0.55	1.50	0.59	1.50	0.63
*Melaleuca viridiflora* (niaouli)	**1.00**	2.00	1.13	**1.00**	0.53	**1.00**	0.63	**1.00**	0.53	**0.75**	***0.42***	2.00	1.17
*Melissa officinalis* (lemon balm)	**1.00**	1.50	0.84	**1.00**	0.53	**1.00**	0.63	**1.00**	0.53	1.50	0.84	**1.00**	0.58
*Pogostemon patchouli* (patchouli)	**1.00**	1.25	0.70	6.00	3.19	**1.00**	0.63	2.00	1.06	**1.00**	0.56	1.25	0.73
*Santalum album *(sandalwood)	**1.00**	**0.50**	***0.28***	**1.00**	0.53	**1.00**	0.63	**1.00**	0.53	**1.00**	0.56	**1.00**	0.58
*Styrax benzoin *(benzoin)	2.00	**1.00**	***0.31***	2.00	0.56	8.00	3.00	**1.00**	***0.28***	**1.00**	***0.31***	**1.00**	***0.33***
*Syzygium aromaticum* (clove)	**0.50**	**1.00**	1.06	**0.75**	0.77	**1.00**	1.13	**0.75**	0.77	**1.00**	1.06	**1.00**	1.08
*Thymus vulgaris *(thyme)	**1.00**	4.375	2.46	**1.00**	0.53	2.00	1.25	**1.00**	0.53	1.50	0.84	**1.00**	0.58
*Vetiveria zizanioides *(vetiver)	**1.00**	**1.00**	0.56	**1.00**	0.53	**1.00**	0.63	**1.00**	0.53	**1.00**	0.56	1.50	0.88

*∗*Noteworthy antimicrobial activity highlighted in **bold.**

†Synergistic interactions highlighted in ***bold italics.***

**Table 4:** Essential oil cytotoxicity alone and in combination with carrier oils (% mortality).

**Essential oil**	**Cytotoxicity alone**		***Aloe*** ***vera***			***Calendula*** ***officinalis***			***Hypericum*** ***perforatum***			***Persea*** ***americana***			***Prunus*** ***armeniaca***			***Simmondsia*** ***chinensis***		
	**24 hrs**	**48 hrs**	**24 hrs**	**48 hrs**	Σ**FIC**	**24 hrs**	**48 hrs**	Σ**FIC**	**24 hrs**	**48 hrs**	Σ**FIC**	**24 hrs**	**48 hrs**	Σ**FIC**	**24 hrs**	**48 hrs**	Σ**FIC**	**24 hrs**	**48 hrs**	Σ**FIC**
*Cananga odorata* (ylang ylang)	77.36^*∗*^	94.15	4.60	10.20	0.24^†^	6.95	12.44	**0.29**	5.54	13.59	**0.24**	0.67	8.96	**0.22**	0.94	17.03	0.91	1.74	11.16	**0.29**
*Cinnamomum zeylanicum *(cinnamon)	97.68	97.68	2.58	5.56	**0.13**	76.95	87.92	2.00	25.71	64.94	1.16	0.00	40.67	1.00	100.00	100.00	${\underline{5.33}}^{‡}$	10.67	40.86	1.06
*Citrus bergamia* (bergamot)	94.29	96.95	78.58	89.48	2.11	66.36	84.68	1.93	79.16	91.48	1.63	0.49	19.73	**0.48**	3.75	19.27	1.03	4.63	23.17	0.60
*Citrus reticulata* (mandarin)	73.62	90.96	6.76	13.03	**0.31**	41.54	64.35	1.49	9.93	20.26	**0.37**	0.57	13.12	**0.33**	0.63	19.30	1.04	11.94	100.00	2.62
*Commiphora myrrha *(myrrh)	76.99	92.99	3.86	9.66	**0.23**	9.33	18.05	**0.42**	4.06	8.06	**0.15**	12.61	83.33	2.07	1.41	35.83	1.92	3.32	29.21	0.76
*Cymbopogon citratus *(lemongrass)	100.00	100.00	9.49	21.42	**0.50**	71.43	82.38	1.86	19.52	44.01	0.78	40.43	54.90	1.34	1.10	27.54	1.46	6.27	90.61	2.33
*Cymbopogon martinii *(palmarosa)	93.32	94.41	3.64	15.40	**0.37**	2.95	13.65	**0.31**	10.37	30.12	0.54	0.00	18.19	**0.45**	1.54	28.93	1.55	100.00	100.00	2.60
*Eucalyptus globulus* (eucalyptus)	15.48	77.66	0.00	4.00	**0.10**	0.98	8.31	**0.20**	2.57	7.27	**0.14**	0.72	30.86	0.80	1.32	5.19	**0.28**	2.06	9.45	**0.26**
*Helichrysum italicum *(immortelle)	20.41	40.09	2.88	10.55	**0.33**	0.47	23.81	0.72	2.89	7.75	**0.20**	5.98	39.86	1.27	1.93	14.01	0.85	7.78	90.00	2.99
*Kunzea ericoides* (kanuka)	28.08	82.94	4.00	16.16	**0.40**	3.36	6.06	**0.14**	0.52	2.64	**0.05**	3.84	5.16	**0.13**	0.94	13.37	0.72	3.28	72.39	1.94
*Laurus nobilis* (bay)	31.89	42.92	0.53	8.84	**0.27**	4.35	9.18	**0.27**	7.23	9.78	**0.24**	2.34	7.86	**0.24**	9.89	23.98	1.43	1.56	5.11	**0.17**
*Lavandula angustifolia* (lavender)	54.52	94.14	0.46	4.32	**0.10**	3.75	9.64	**0.22**	3.42	6.42	**0.12**	75.76	75.76	1.87	0.62	16.83	0.90	3.55	67.87	1.77
*Leptospermum scoparium* (manuka)	95.49	98.82	10.24	59.70	1.40	4.17	8.64	**0.20**	5.37	7.81	**0.14**	1.32	13.46	**0.33**	7.01	32.85	1.75	2.68	61.80	1.59
*Litsea cubeba *(may chang)	99.58	99.58	4.37	23.98	0.56	2.97	27.97	0.63	0.52	5.51	**0.10**	1.26	41.26	1.01	0.58	12.98	0.69	2.45	72.22	1.86
*Melaleuca alternifolia* (tea tree)	71.17	94.20	0.46	3.29	**0.08**	4.57	6.60	**0.15**	4.81	7.34	**0.13**	100.00	100.00	2.47	0.29	6.74	**0.36**	0.00	15.83	**0.41**
*Melaleuca viridiflora* (niaouli)	33.43	61.32	1.05	4.23	**0.11**	4.22	7.89	**0.20**	3.66	7.21	**0.15**	0.91	3.57	**0.10**	1.10	11.80	0.66	0.56	56.28	1.63
*Melissa officinalis* (lemon balm)	92.31	96.25	6.14	15.95	**0.38**	2.59	5.05	**0.12**	6.15	8.21	**0.15**	5.93	16.63	**0.41**	2.19	13.93	0.74	1.17	22.40	0.58
*Pogostemon patchouli* (patchouli)	88.49	95.33	7.77	27.13	0.64	3.81	19.16	**0.44**	8.21	27.07	**0.49**	0.55	7.97	**0.20**	8.71	29.02	1.55	2.18	38.05	0.99
*Santalum album *(sandalwood)	96.12	99.13	1.59	8.61	**0.20**	4.84	11.73	**0.27**	4.20	17.51	**0.31**	32.11	67.00	1.64	3.59	38.56	2.05	0.00	32.64	0.84
*Styrax benzoin *(benzoin)	16.40	68.66	5.28	8.98	**0.23**	10.91	28.28	0.70	9.73	17.99	**0.36**	13.17	35.87	0.96	31.53	43.99	2.44	11.84	60.62	1.70
*Syzygium aromaticum* (clove)	98.88	99.46	4.33	12.97	**0.30**	99.42	99.42	2.25	98.93	98.93	1.76	100.00	100.00	2.44	78.61	100.00	5.32	4.20	32.63	0.84
*Thymus vulgaris *(thyme)	100.00	100.00	86.32	97.11	2.28	54.61	70.39	1.59	99.48	99.48	1.76	7.49	20.52	**0.50**	84.68	88.63	4.71	52.00	85.22	2.19
*Vetiveria zizanioides *(vetiver)	11.37	27.43	2.52	4.57	**0.17**	4.93	10.45	**0.37**	3.51	8.50	**0.26**	0.37	98.96	3.73	0.62	9.05	0.60	0.67	7.74	**0.30**

*∗*Percentage cytotoxicity above 50 is considered toxic; below 50 is considered nontoxic.

† synergy highlighted in bold.

‡ antagonism shown with underline.

## Data Availability

The data used to support the findings of this study are available from the corresponding author upon request.

## Supplementary Materials

Supplementary Materials Essential oil selection based on popularity and antimicrobial activity reported in our previous study.
