# Supplementary material for: The Influence of Carrier Oils on the Antimicrobial Activity and Cytotoxicity of Essential Oils
Source: Evid Based Complement Alternat Med. 2019 Jan 14;2019:6981305. doi: 10.1155/2019/6981305 (PMC6348851; doi:10.1155/2019/6981305)
Supplement: Supplementary Materials — Essential oil selection based on popularity and antimicrobial activity reported in our previous study. [file 6981305.f1.docx]

**Supplementary data**

**The influence of carrier oils on the antimicrobial activity and toxicity of essential oils**

**Ané Orchard^a^, Sandy F. van Vuuren^a*^, Guy Kamatou^b^, Alvaro M. Viljoen^b,c^ , Namita Patel^a^ and Patricia Mawela^a^**

^a^ University of the Witwatersrand, Faculty of Health Sciences, Department of Pharmacy and Pharmacology, 7 York Road, Parktown 2193, South Africa

^b^ Tshwane University of Technology, Faculty of Sciences, Department of Pharmaceutical Sciences, Private Bag X680, Pretoria 0001, South Africa

^c^ SAMRC Herbal Drugs Research Unit, Department of Pharmaceutical Sciences, Private Bag X680, Pretoria 0001, South Africa

***** Correspondence author

Sandy van Vuuren, Department of Pharmacy and Pharmacology, University of the Witwatersrand, 7 York Road, Parktown 2193, Johannesburg, South Africa.

E-mail: sandy.vanvuuren@wits.ac.za

Phone: +2782-743-1125

Fax: 0865534737

Essential oil selection based on popularity and antimicrobial activity reported in our previous study

| **Popularity** | **Noteworthy** | **Moderate** | **Poor** |
| --- | --- | --- | --- |
| *Cananga odorata* (ylang ylang) | *Cymbopogon citratus* (lemongrass) | *Helichrysum italicum* (immortelle) | *Cananga odorata* (ylang ylang) |
| *Cymbopogon citratus* (lemongrass) | *Laurus nobilis* (bay) | *Leptospermum scoparium* (manuka) | *Citrus bergamia* (bergamot) |
| *Eucalyptus globulus* (eucalyptus) | *Pogostemon patchouli* (patchouli) | *Melaleuca viridiflora* (niaouli) | *Citrus reticulata* (mandarin) |
| *Lavandula angustifolia* (lavender) | *Syzygium aromaticum* (clove) | *Melissa officinalis* (lemon balm) | *Kunzea ericoides* (kanuka) |
| *Melaleuca alternifolia* (tea tree) | *Vetiveria zizanioides* (vetiver) | *Styrax benzoin* (benzoin) | *Lavandula angustifolia* (lavender) |
| *Thymus vulgaris* (thyme) | *Cinnamomum zeylanicum* (cinnamon) | *Thymus vulgaris* (thyme) | *Melaleuca alternifolia* (tea tree) |
|  | *Commiphora myrrha* (myrrh) | *Eucalyptus globulus* (eucalyptus) |  |
|  | *Cymbopogon martinii* (palmarosa) |  |  |
|  | *Litsea cubeba* (may chang) |  |  |
|  | *Santalum album* (sandalwood) |  |  |

*Antimicrobial activity as determined by Orchard et al. (2017)
